# Digital phenotyping and the (data) shadow of Alzheimer’s disease

**DOI:** 10.1177/20539517211070748

**Published:** 2022-01-11

**Authors:** Richard Milne, Alessia Costa, Natassia Brenman

**Affiliations:** 1 Engagement and Society, Wellcome Connecting Science, Hinxton, UK; 2 Cambridge Public Health, University of Cambridge, Cambridge, UK; 3 Department of Sociology, Goldsmiths, University of London, London, UK

**Keywords:** Data shadow, digital phenotype, data double, digital health, ageing, Alzheimer’s disease

## Abstract

In this paper, we examine the practice and promises of digital phenotyping. We build on work on the ‘data self’ to focus on a medical domain in which the value and nature of knowledge and relations with data have been played out with particular persistence, that of Alzheimer’s disease research. Drawing on research with researchers and developers, we consider the intersection of hopes and concerns related to both digital tools and Alzheimer’s disease using the metaphor of the ‘data shadow’. We suggest that as a tool for engaging with the nature of the data self, the shadow is usefully able to capture both the dynamic and distorted nature of data representations, and the unease and concern associated with encounters between individuals or groups and data about them. We then consider what the data shadow ‘is’ in relation to ageing data subjects, and the nature of the representation of the individual’s cognitive state and dementia risk that is produced by digital tools. Second, we consider what the data shadow ‘does’, through researchers and practitioners’ discussions of digital phenotyping practices in the dementia field as alternately empowering, enabling and threatening.

## Introduction

In this paper, we draw on work with researchers and developers engaged in digital health to explore how digital phenotyping represents the ageing body, and, further, how these representations act as entities in their own right. We build on sociological and anthropological work on the data self and focus on a medical domain in which the value and nature of knowledge and relations with data have been played out with particular persistence, that of Alzheimer’s disease research.

As a leading cause of ill-health in high-income countries, dementia has been the focus of considerable policy and research attention since the mid-1980s. Much of this attention has concentrated on Alzheimer’s disease, the most common cause of dementia. As is the case across biomedicine, the collection, sharing and analysis of large volumes of data is seen as central to the future of Alzheimer’s disease research. Such data, derived from genomics, electronic health records and increasingly from digital sources, is intended to enable the detailed characterisation of individual patients that lies at the heart of ‘precision’ medicine. While Engelmann (2020) argues that there is both an epistemic and sociological naivety to the belief that clear disease will emerge from the mining of data, it remains a powerful driving impetus, particularly in fields like Alzheimer’s disease research in which the connections between the normal and the pathological, the biological and the clinical, remain profoundly contested (cf Lock, 2013).

A shift in Alzheimer’s disease research and in diagnostic criteria in the last two decades has placed biomarkers at the centre of what constituted ‘disease’ and created a drive to better understand the ‘pathway’ of biomarker change over time. It has driven a focus on the thresholds at which an individual can be detected as moving between stages on this pathway from ‘normal’ to ‘biomarker positive’, or from healthy to preclinical, prodromal and on to symptomatic dementia. Research increasingly focuses on understanding the pathology, natural history and epidemiology of the disease to prevent or delay the process of cognitive decline. The hope is that this move to prevention will counter the long-standing status of the field as a ‘graveyard of drug development’ (Hawkes, 2016). It frequently involves, however, the identification of people deemed to be at ‘high-risk’, those who might be most eligible for clinical trials (Milne, 2018).

Since the identification in 1993 of the first gene associated with susceptibility to Alzheimer’s disease, the ApoE e4 allele, an enduring bioethical debate has taken place about whether and how information about risk states should be made available to individuals, and what the consequences would be of doing so (Post, 1996). As Alzheimer’s disease research and in many cases clinical care have become increasingly dominated by assessments of the biological state of the brain (Lock, 2013), these debates have been taken forward into discussions around the use of biomarkers such as beta-amyloid or tau to assess an individual’s risk of future dementia (Karlawish, 2011; Schicktanz et al., 2014). This has spurred a growing body of work on the experience of living with information about Alzheimer’s disease risk (Largent et al., 2020; Milne et al., 2018). It has also, however, prompted critical commentary on the constraints of biology-centred representations of current and future health that marginalise the collection or use of information about environmental exposures or lived experiences (Brayne and Kelly, 2019; Leibing and Schicktanz, 2020; Lock, 2013).

Questions about the adequacy of representation and the experience of a life lived in relation to information about future health are made all the more pressing and relevant by the emergence of ‘digital phenotyping’ in medical research and accompanying promissory narratives (Birk et al., this issue). Digital phenotyping approaches are based around the fundamental promise that an individual’s experience of health, ‘is expressed in the digital traces that a person leaves behind’ (Birk and Samuel, 2020: 1873). In the context of psychiatry, leading proponents of the digital phenotyping approach position it as a response to the concern of many psychiatrists that the field was moving from being ‘brainless’ to ‘mindless’ (Insel, 2017: 1215). In his *JAMA* article setting out his programme for digital phenotyping as ‘a new science of behaviour’ (2017), Insel, the former director of the US National Institute of Mental Health, argues for acknowledgement of the limitations of the growing dominance of a focus on the biological correlates of mental health. He describes a risk that recent psychiatric research and diagnostic practice have been dominated by genomics, pharmacology and neuroscience at the expense of behavioural assessments and detailed clinical interactions. Instead, Insel and other exponents of a digital phenotyping approach suggest that it enables researchers and clinicians to use portable tools and informatic techniques to capture the ‘extended mind’ (Raballo, 2018).

Digital phenotyping approaches are based around either active user engagements with tests or assessments, or on ‘passively’ collected data generated in everyday life and engagements with the world. The central promise is ‘the possibility of continuous measurements. Use of apps, phone calls, typing speed, and voice features can be monitored unobtrusively every second over a lifetime, with real-time algorithms checking for alarming transformations’ (Ebner-Priemer and Santangelo, 2020: 298). As with the wider field of psychiatry, neurodegenerative disorders and dementias including Parkinson’s (Trister et al., 2016) and Alzheimer’s disease (Kourtis et al., 2019) are important sites of hope and promissory investment.

The development of digital phenotyping tools draws into focus the intersection between data practices of disease detection and those associated with the extraction of the ‘behavioural surplus’ of surveillance capitalism (Zuboff, 2019). These come together explicitly in work from companies including Microsoft, Google and Intel. Microsoft, for example, has explored the possibility of using search engine data to develop ‘web search digital phenotypes’ that can be used to detect neurodegenerative disorders (White et al., 2018)). Patents for the Google Home device meanwhile describe a possible use of the device to analyse ‘the unique signatures of the occupants’ for patterns indicative of Alzheimer’s (Fadell et al., 2018: 252). These ‘unique signatures’ draw discussion of digital phenotyping into dialogue with the wider body of work on the configuration and exploitation of the ‘digital subject’ (Goriunova, 2019). In the following section, we draw on this literature to consider the forms of representation and the relation between individuals and data described in the development of digital tools for the assessment of cognition.

## Doubles and shadows – life with data

The idea that distinctive features of our identity might be reassembled using the digital tracks and traces of our lives – our ‘unique signatures’ – has generated a rich and continuing conversation on the formation of digital subjects and its consequences. Schüll highlights the ‘creative vocabulary’ (2018: 35) introduced by scholars aiming to capture the intensive datafication of life in western societies. These include the use of terms such as ‘data doubles’ or data selves to describe emergent and temporary virtual/informational profiles that are aggregated from different data sources, sliced and circulated and through which selves become both objects and subjects of power in digital worlds (Douglas-Jones, 2021; Green and Svendsen, Haggerty and Ericson, 2000; Lupton, 2019). Thus, confronted by an advertising company’s elaboration of the use of data to target a specific consumer – herself – Goriunova asks

“What exactly is this digital entity that she identified as me? What relation does it have to me? How do I relate to it? How is it able to stand in for me and construct a me that attracts advertisements and thus alters me, while still being reliant on my activity? How is it produced outside of my awareness, mobilized, and recruited?” (2019: 126)

The mapping of persons to digital traces captured over time produces a particular kind of subject formation. However, as Goriunova here suggests, this digital entity acts and alters the subject to whom it relates – as Lupton puts it ‘people and their data make each other’ (Lupton, 2018: 5). In this, data doubles echo the discussion of Frank, who draws attention to the multiple images and codings through which the body is doubled and redoubled in contemporary medicine, such that the ‘image on the screen becomes the “true” patient’ and ‘initial certainty of the real (body) becomes lost in hyperreal images that are better than the real body’ (in Nettleton, 2004: 669).

Lupton, engaging with the concept of the data double in the case of health data, describes how such abstracted ‘data-doubles’ both categorise and identify ‘at-risk’ individuals, and become materially forceful, ‘feeding back information to the user and encouraging the user’s body to act in certain ways’ (Lupton, 2012: 237). As she continues, the data double is part of ‘a continual loop of the production of health-related data and response to these data’ (Lupton, 2012: 237). In her work with members of the quantified self-movement, ‘pioneers in the art of living with and through data’ (Schüll, 2018: 35) who represent the contemporary apogee of self-tracking and monitoring practices, Schüll captures how data becomes part of a ‘loop of reflexive recomposition’ (2018: 35) and digital tools provide self-trackers the freedom to engage in projects of self-transformation. This ‘loop’ of (self-) representation and transformation is both recognised and valued by those involved in the development and capitalisation of data-driven tools. It is central to the premise of fitness and ‘wellness’ devices that aim to encourage reflexive interactions with the data self, whether for health or wealth (Douglas-Jones, 2021; Lupton, 2019).

In this paper, we explore the nature of the ‘data self’ using the metaphor of the ‘data shadow’. The data shadow refers to the counterpart to the individual produced through everyday interactions and encounters with data collection and storage (Zook et al., 2004). Such ‘thick data shadows’ are not ‘just a way of describing data itself, and our increased prowess in measuring, mapping, analyzing, and visualizing, but a meme that speaks to and produces new ways of establishing truth’ (Graham and Shelton, 2013: 257). However, as Leonelli and colleagues point out, the concept also draws attention to ‘an ambiguity and a strategic relationality to shadowing processes that parallels the relational nature of data and the multiplicity of motives, goals, and conditions through which data may be construed as (in)significant, partial or complete, (un)intelligible, or (in)accessible’ (Leonelli et al., 2017: 194). This focus draws critical attention to how representational apparatuses are assembled, how patterns of illumination and obscurity are authorised and legitimised, how and why some aspects of the data self are drawn into the light, seen as ‘available, portable, and/or meaningful’ (2017: 194) and other data made ‘missing, unavailable, or invisible’ (2017: 191), and how this changes over time (cf McGoey, 2017).

First, we suggest that the idea of the data shadow extends discussion of the representational nature of the data object, following Goriunova (2019) in complicating representations of data subjects as 1:1 depictions of persons or subject positions and emphasising the strategic relationality highlighted by Leonelli et al. (2017). Although Gorinuova treats ‘data doubles’ and ‘shadows’ as interchangeable notions of the indexical digital subject, we argue that the relational nature of shadows offers possibilities that have not yet been explored. Thus, while the notion of ‘doubling’ concentrates attention on duplication and replication, the nature of the shadow usefully and intuitively captures the dynamic and distorted (cf Green and Svendsen, this issue) nature of data representations. It draws attention to the circumstances of the production of the data self: the relations between the properties of an object or figure, how that figure relates to a light source, the ground against which it stands and on which the shadow is cast, and the place of the observer. It is this configuration, and the epistemic strategies that define it that enables a shadow to be produced.

Second, the shadow has a cultural resonance that gives the notion of the data shadow value beyond representational concerns. As an artistic, literary and cinematic trope, the shadow has value for its ability to capture the unease and concern associated with encounters between individuals or groups and data about them (Stoichita, 1997). In these contexts, the shadow is not simply a representation of an absent subject so much as an entity in its own right, a ‘reality to your consciousness‘ (Bergson, 1913: 54). As such, it can form its own object of study, a menacing other rather than a represented self. Consequently, ‘we produce our own data shadow, but do not have full control over what it contains or how it is used to represent us’ (Zook et al., 2004: 169). Such shadows have value and can be traded in networks of exchange, creating situations in which a subject may feel ‘over-shadowed’ by circulating information about them – for example, as they attempt in the contexts of employment, finance or insurance. These entities are not simply archives capable of revealing ‘hidden patterns of action at play in our day-to-day lives’ (Schüll, 2018: 5). Our ‘othered’ data selves here pose potential threats to the life chances and choices of individuals, including when they are considered to be predictive of future problems (Eubanks, 2018; Lyon, 2014; Ruppert, 2012).

Individual lives are thus not simply captured in data, but lived in relation to these data and the futures they ‘foreshadow’. In this sense, the concept of the data shadow allows us to interrogate how access to services, from healthcare to insurance, is shaped by the future selves cast forwards by data. The absence of such foreshadowing, though, can also be problematic. As Beer puts it ‘When we are informational persons – that is to say, when we have become our data – the deletion of our data amounts to the erasure of our identities’ (Beer, 2021: 390). Elaborating on this, the lack of a data shadow may limit access to future-oriented domains such as insurance. The ‘active’ life of our data shadows, and the material consequences of their absence thus emphasise both the consequences of living in relation to and without our data self.

In juxtaposing such representational and material, ‘virtual’ and ‘vital’ elements of the data shadow, we do not intend to sharpen the distinction between the digital and lively, biological processes – between knowledge of life and life itself. Such a distinction is far from clear, perhaps least so amongst those people producing the tools and knowledge to phenotype complex neurological conditions such as dementia. Rather, we aim to explore the different relationships that are forged between living subjects and the informational traces of ageing bodies and minds. In this way, we are more interested in the messy relationships between knowledge of life and life itself; and in digital phenotyping as only one iteration of the data shadow, with its own particular qualities, distortions and effects.

In the following sections, we move on to extend and illustrate our discussion through our empirical data. We consider what the data shadow ‘is’ in relation to ageing data subjects, and the nature of the representation of the individual’s cognitive state and dementia risk that is produced by digital tools. Second, we consider what the data shadow ‘does’, through researchers and practitioners’ discussions of digital phenotyping practices in the dementia field as alternately empowering and threatening.

Our discussion draws on two research studies. The first, an empirical study of ethical challenges associated with digital detection in dementia, involves work with both domain experts, reported here, and older adults around their use of, and experience with, digital health. The work reported here consisted of a domain mapping of academic and commercial activity and research in the field, incorporating published academic and patent literature and presentations of tools in websites, company webinars and press releases and conference presentations, covering 30 tools under commercial development. This mapping was followed by 26 semi-structured interviews in 2019 and 2020. Participant was drawn from companies involved in the development of digital tools (*n* = 7), academic neuroscience and data science researchers (*n* = 8), clinicians in neurology or old age psychiatry (*n* = 3) and policy and research officers working in non-profit organisations involved in developing or funding digital phenotyping tools for dementia (*n* = 9). Participants were based in the UK, Europe and North America. Interviews asked about respondents’ hopes and expectations around the tools they were involved in developing, their concerns and their awareness of ethical considerations related to the use of digital tools for the early detection of cognitive decline.

The second study involved three focus group discussions held in the UK in 2020. Ten UK-based researchers participated, drawn from academic neuroscience and data science, clinical research, industry and clinical old-age psychiatry and neurology. All were involved in the development of a large project aimed at the development of markers of Alzheimer’s disease progression for use in clinical trials. While not all were directly involved in the development of digital tools, the project as a whole aimed to incorporate these into clinical trial practice. These focus groups again concentrated on expectations and concerns around the future role of digital tools in the assessment of cognitive decline.

In the following sections, we explore how this data can provide insight into how the value of digital phenotyping is being imagined, and the challenges associated with this. We draw on the perspectives of interviewees who are, in the main, involved in articulating and giving impetus to the possible futures of digital health. As such, we are aware that this focus on hopeful narratives, often oriented towards the creation of promissory value, creates our own ‘data shadows’ and absences (cf Leonelli et al., 2017), not least those associated with alternative ways of seeing, doing and living Alzheimer’s disease (Leibing, 2014).

## Representing cognition: Casting shadows

In this section, we explore how our interviewees, many of whom are deeply invested in the development of digital phenotyping and the scientific and clinical exploitation of the data shadow, conceptualise the promise of their approach and the value, nature and status of the representations they are involved in producing. We describe how, in interviews, publications and corporate material, the promise of digital phenotyping tools is established through three closely related discourses of epistemic value. These discourses emphasise uniqueness and the detail of digital representations of individuals; extension or the ability to track an individual over time; and ecological validity, or the possibility of ‘real world’ measurement. These discourses, we argue, allow us to understand the strategic relationality of digital phenotyping’s data shadow.

Interviewees and other proponents of digital approaches to capturing cognitive and behavioural states claim that these offer the possibility of capturing data that is distinctively representative of an individual. As a consequence, they suggest, such tools are able to depict an individual’s brain health in a manner that is both potentially both more profound and more able to matter to people. Thus, for example, interviewees described how the use of digital devices is unique to each individual and thus uniquely identifying. As one interviewee put it ‘the way that people interact with their phone, is like a fingerprint for them’ (staff member, non-profit organisation). This fingerprint generated in interaction with devices, in turn, has scientific and potential clinical value for developers in excess of its uniquely identifying capacity; for example, through its use to generate a ‘cognitive fingerprint’ (researcher, non-profit). As another interviewee described, such fingerprints may be based on the aggregation of multiple digital streams that capture the multiple aspects of a ‘neurological condition … like slight behavioural changes, executive function changes, the syntax of your language’ (Researcher, non-profit).

As in wider discussions of digital phenotyping (Birk and Samuel, 2020), the ability of digital representations to act as measures of an individual’s brain health is not universally accepted amongst our respondents, as we discuss below. However, for interviewees involved in elaborating the promise of digital approaches, only part of their value lies in their individualisation and a persistent ‘fingerprint’. In fact, the value of this representation lies in its instability. Unlike its physical counterpart, the digital ‘fingerprint’ is mutable and changes over time. What the fingerprint ‘is’ in this case is defined by what it can technically ‘do’, record and follow change, track the process of an individual becoming different from themselves and extrapolate from this to infer future health. For our respondents, it is this focus on individual fluctuation and change that distinguishes digital phenotyping:

“Digital tools enable you to detect change, and will put more emphasis on the role of this fluctuation and this decline before you actually need to reach a threshold to get any intervention.” (Company researcher)

The move away from thresholds and towards subliminal fluctuations in a continuously reproduced image of the self has consequences for the forms and content of the data that make up this picture. As other interviewees emphasised, it may be that a critical feature of digital phenotyping is not simply the accumulation of information from passively collected data. Instead, it is in the extraction of value through inference, in a manner that echoes – and indeed explicit references the wider ‘digital exhaust’ (Hirschtritt and Insel, 2018). As one interviewee put it:

“We know language changes in later stages without being detected. So, that’s one of the reasons why we’re looking that earlier. … But then … the literal data and words you say is not what we care about. It’s what insight that gives us into the change that’s going on in your brain” (Ethics officer, non-profit)

Another senior company interviewee described how such functional measures – and their algorithmic interpretation – can provide truly ‘personal’ assessments:

“We have a beta version that we are testing internally … and the data is fascinating because it shows that it’s absolutely personal, for myself I can see sleep has a huge impact on my performance. Some other colleagues that do this, they can see that the days that they have physical exercise … you can see a huge improvement in how their brain capacity improves. We have done this in a small group of people over a six month period, we’re basically training the AI to understand what factors we need to consider.” (CEO, UK-based digital health company)

This promise of better representations of an individual’s world, produced by repeated measurement over time, is reflected in the Delphic corporate slogan of the leading digital cognitive testing company Altoida to ‘Know Thyself’, and in the motivations of researchers working across the field. As one interviewee described:

“It was our understanding that some of these people were saying that they had subjective cognitive complaints, saying that in their opinion their cognitive ability was declining, but on their standard tasks they were doing just fine, so a clinician couldn’t say that they had any kind of deficits or impairments … so we tried to understand why these people were actually talking about their cognition declining even though the tests that we were using weren’t picking up on that, so we just tried to make tests as close to real life” (Researcher, non-profit)

For this interviewee, digital tests have the potential to provide more accurate representations of cognitive state that may more closely align with lived experience and ‘real life’. This extract, and its emphasis on individuals’ experiences of decline, highlights the temporal ambitions of those developing digital tools, to track change over time. It also, however, introduces a third key feature of digital phenotyping that draws attention to *situated* change. The unique fingerprints described by our respondents and their change over time are conceptualised as reflecting an individuals’ ‘real life’ engagement with the world.

Being able to track the process of becoming different is part of the possibility of a new form of knowledge. As one company CEO described to us how:

““I think what’s important here is that we have no good biomarkers for the brain … nothing that really is measuring functional capacity, nothing that’s, we’re measuring illness but we’re not really measuring the impact that it’s having on someone’s ability to function, to use their brain to solve a task and live a healthy life, so that’s important” (CEO, US-based digital health company)

For this individual, articulating their vision for the technology and its potential, digital phenotyping allows the ‘real measurement’ of the impact of illness through an individual’s lifetime. For them, this remedies the limits and blind-spots of existing measures, moving the field towards capturing the lived experience of ‘using the brain’ to ‘live a health life’.

This commitment to continuous measurement in ‘real life’ forms the core of what the developers and researchers we interviewed refer to as ‘ecological validity’, a central epistemological premise of the digital phenotyping enterprise. For actors in the field, this claim to ecological validity posits that digital tools’ ability to provide access to the ‘real world’ makes their data representations qualitatively different from existing ways of seeing and ‘doing’ Alzheimer’s disease. As one interviewee put it, ‘it tells us about what it is to be a human being with this brain interacting with the world.’ (clinical researcher). Another described how:

“Technology can give you the real-world scenario, continuous data, understanding a little bit more about how, really, people function and behave and how their condition is.” (Company researcher)

As this researcher makes clear, the promise of technology for them is both temporal, in the form of ‘real-time’ continuous data, and ethological, in the form of real behaviour. This suggestion, repeated across interviews, that digital phenotyping captures how people ‘really’ function recapitulates questions in the wider psychology (and sociology) literature of the relationship between experimental evidence produced in laboratory settings and behaviours ‘in the wild’ (Cicourel, 1982, 1996; Orne, 1962). Indeed, the transferability of evidence beyond the laboratory or the clinic has been a persistent question for the neuropsychological research on which many digital measures of cognition build (Chaytor and Schmitter-Edgecombe, 2003; Kvavilashvili and Ellis, 2004). Those working on digital tools thus suggest the limits of measures and tasks that ‘require subjects to perform a task outside of the context of everyday behaviour’ (Dagum, 2018: 1), and emphasise the value of collecting data using smartphones “in a natural environment” (Dagum, 2018: 2). Dagum’s discussion here closely Insel’s perspective on the development of the ‘new science of behaviour’ – and indeed, the two co-founded the mental health and cognitive testing company Mindstrong (Reardon, 2017).

In our study, researchers using virtual and augmented reality approaches to assessment particularly emphasised the possibility of generating representations of cognition created by ‘making something real life’ (company researcher). They suggest that digital approaches offer the possibility of a wider shift towards new ways of seeing:

“I kind of get the sense that sometimes in medicine it’s just, you know, checking the box. They’ve a cognitive assessment. And they don’t really care what the cognitive assessment is, they’re more interested in the biology of the disease, because that’s what the treatment is going to have an effect. That’s why it’s almost as if seeing the pathology and the disease go down is what you want to see. And if that has a cognitive effect, then fine. Good. It’s almost always been secondary to the cognitive change has always been secondary to the biomarker change. And I think that’s shifting now.” (Researcher, non-profit)

The transition this researcher describes and urges, away from a biomarker-driven approach to ‘seeing the pathology go down’ towards a focus on cognitive change is reflected in the approaches of a number of companies and projects operating in the field – the digital health companies Winterlight and MyndYou, for example, echo the chief scientific officer of Applied Cognition (Dagum, 2018) in describing how their analyses of voice data give ‘ecological’ measurements of cognitive state.

As the extracts above suggest, the development of digital tools is taking place amid an existing marketplace of approaches to representing the ageing brain. In positioning their field respondents suggest distinctions or even sequences in these approaches. The future applications of digital phenotyping they describe explore relations of commonality, complementarity and conflict between digital and biological ways of knowing and representing brain health. For some interviewees, the shadows cast by digital and biological data were complementary, and associated with different aspects of disease – as one put it:

“I mean digital data can measure definitely your activities of daily living. So I think it’s good for the people who are not I mean, who are a little bit, they’re not very serious who are slowly going towards maybe the cognitive impairment phase. And then, yeah, then the dementia phase. So for early disease prediction I think this is the case that these patients are given the digital data. But if you think that a person [has dementia] you have to somehow take brain images of the patient. That cannot be done with a digital readout, because *to really see if this patient has dementia or not, then you have to look into the brain of the person* and you have to see that something has happened.” (Clinical researcher, emphasis added)

For this interviewee, the data shadow produced by digital phenotyping both ‘definitely’ measures daily life, and *casts forward* the future health of individuals ‘slowly going towards’ cognitive impairment and dementia. The data shadow’s ability to represent, however, is partial – it cannot sufficiently illuminate the *current* state of the brain and, in order to align the clinical data shadow with the disease model articulated in dominant diagnostic definitions of disease, has to be accompanied by measurement of the changes in biological markers of brain health.

For the interviewee above, the representation of the ageing body produced by digital phenotyping is complementary to the biological. For others, the biological model of Alzheimer’s disease meant that the shadows cast by digital phenotyping were emphatically the ‘wrong’ type of picture. As one senior clinical researcher put it:

“I think digital biomarkers are a new way of measuring the wrong thing … Alzheimer’s disease is a brain disease, it is not a cognitive disorder. If you want to measure the brain disease, you *measure it directly* with biomarkers and imaging, *you do not measure it on cognition*.” (Clinical researcher, emphasis added)

In criticising digital approaches, this researcher negates the claim to ecological validity – reducing it to an outmoded focus on cognition, and suggesting that the data captured by digital phenotyping is distorted and unhelpful. They contrast this misguided approach to measurement with that focus on ‘brain disease’, suggesting that the data produced by assessments such as brain imaging allow *direct* access to the causes of disease. This speaks to the epistemic and political commitments that shape the ground on which data shadows are cast: the picture of ‘brain disease,’ as opposed to ‘cognitive disorder’ underpins the project of identifying early biological markers, in order to develop drugs that make Alzheimer’s potentially treatable, even before cognitive signs of dementia emerge. Such approaches are often tied to the development of therapies, in which the object to be illuminated through a clinical assessment may be determined by the target of a drug. The context in which data shadows are produced is thus full of coexisting and sometimes competing for projects and perspectives.

## The data encounter: Living with shadows

As introduced above, digital phenotyping produces data representations that are both contemporaneous and predictive, existing alongside the user while suggesting possible futures. As a result, encounters with data shadows involve both current and future health and illness. This encounter with foreshadowed illness, the possibilities it offers and the harms it may cause, have been a repeated site of ethical contestation in the Alzheimer’s disease field (Karlawish, 2011; Post, 1996). In this section, we consider how our interviewees described the nature of this encounter. In doing so, we consider how our respondents conceptualise encounters with data selves as entities that empower and enable, or that threaten.

## The encounter that empowers and enables

The representations discussed above, produced by and through encounters between individuals and digital devices are primarily oriented towards the early detection of cognitive change, and the prediction of an individual’s risk of future dementia. For our respondents, the relationship between an individual data subject and these representations was often couched in terms that emphasise the possibilities afforded by this future orientation. Across digital psychiatry, developers emphasise the ‘biomedical virtue’ of empowering or enabling ‘self-care’ (Pickersgill, 2019). In our study researchers described how datafied representations of cognitive health produced by digital tools could give the subject ‘a window on their self’:

“I think giving some data as a window into their own health, physical and mental, is definitely something we would like to see down the line, whether it’s someone that’s older and healthy or older and has some cognitive or neuropsychological problems.” (Senior academic researcher)

As others described, the ability to provide users with the ability to ‘see through’ this window to look at their data self is the key step in giving them the power to take action:

“we want to be able to collect that data and give a better assessment of where the patient is, and also by making that, visualising that data for the user, not necessarily a patient … we can help them to do things that can help them become healthier” (CEO, UK digital health company 1)

This positive potential of the data shadow to empower recapitulates both the promises of digital psychiatry and, indeed, of digital health more broadly (Lupton, 2012). Thus, another interviewee explicitly situated the goal of their company to use routine digital assessments to enable older adults to maintain their cognitive health firmly within existing data relationships. In this vision, digital cognitive assessments simply fit into the new ways of seeing and being associated with digital health:

“The penetration of Fitbit … for example is really quite advanced into the 60 plus market. So that really is the rise of acceptance of personalised data, and I think that increasingly people do just kind of by second nature understand that this is something that is part of your day to day life.” (CEO, UK digital health company)

For this interviewee the relationship between the data subject and a personalised data self, formerly the domain of the self-tracker, has become commonplace. Our respondents’ elaboration of the relationship in turn attempts to reinforce this idea of digital phenotyping as a quotidian, producing data shadows as unremarkable objects that we live with and alongside. In turn, these data shadows, they suggest, do not simply empower, but enable – they can both contribute to improving health and facilitate wider aspects of life such as employment or insurance. In the Alzheimer’s disease case, this is closely tied to the shift in emphasis towards the early detection of, and intervention in, disease described earlier. As a company researcher described:

“I think a lot of the traditional tools … use thresholds of the output, the score. If you’re below this then you are clinically classified as this whereas, for a lot of people, they might not be below that threshold yet but they will have experienced a lot of change. They would have fallen from where they were previously but according to the standard, traditional, tools they don’t deserve any clinical attention because they haven’t passed that threshold yet.” (Company researcher)

Here, digital tools offer new possibilities for people who experience concerns about changes in their thinking or memory, enabling them to become ‘deserving’ of clinical attention. Such considerations firmly integrate digital phenotyping into existing practices of clinical research, and the spaces of commercial possibility associated with the early detection of disease (cf Dumit, 2012). However, enabling visions of digital phenotyping also connect with, and raises comparable questions to, the role of the data shadow in ‘representing’ or standing in for somebody (cf Goriunova, 2019; Lupton, 2019). One key context for this is insurance, as another interviewee described:

“If I put the clock forward 20 years, I would envisage this. If you’ve got health care insurance, whether it be state or private, part of your annual assessment will be your performance on cognition tests” (Academic researcher)

Here, we see the possibility that the enabling possibilities of the digital phenotyping data shadow derive from their integration into an architecture of insurance-supported healthcare. This vision, elaborated by researchers in both academic and corporate spheres, is evidenced in corporate development models in the field, in which the challenge (and uncertain financial return) of ‘health system’ tools validated to regulatory standards for medical devices is accompanied by ‘lifestyle’ products, which may incorporate the same technical basis, but are oriented towards the more accessible market of health insurance.

Narratives of self-care embedded in insurance-driven healthcare situations situate digital phenotyping firmly within a particular structure of healthcare provision. In that sense, they connect digital health to political discourses of preventive health that shift the burden of action away from the state towards the individual (cf Lupton, 2012) and seek to embed digital tools for cognitive assessment within technology-enabled projects of self-transformation. These visions of empowerment and enablement also, however, emphasise the threat of being absent from data. In addition to being provided with information they can use themselves, in the narratives presented by developers, the ‘measured’ are enabled to access healthcare and insurance services in ways that are not available to the ‘unmeasured’. In this vision of future application, digital phenotyping presents possibilities – as the data shadow becomes supportive of health and facilitative of access to a particular vision of healthcare – but also threatens and restricts, in both its presence and absence.

## The encounter that threatens

The threats to self and to individual autonomy posed by the data shadow were raised by a number of our interviewees and, in many ways, return our analysis towards those concerns raised in the bioethics literature around the return of results in the context of Alzheimer’s disease, and the relationship between discussions of risk and those of affective encounters between individuals and data. Thus, the researcher with whose interview we closed the preceding section continued by elaborating the need for control over one’s data in light of the potential of this data entity to cause harm if allowed to move. As they put it,

“If you let people test your cognition for you, then it probably waived your privacy goodbye very quickly. If you can do that independently then you can claim your data and preserve your privacy.” (Academic researcher)

In this quote, the researcher emphasises that the relationship between self and data should be just that – avoiding intervention or mediation from third parties, with the individual data subject retaining control over both her data and the futures it may foreshadow. However, as interviewees repeatedly raised, this intimate relationship is also one that comes with an existential threat to the data subject herself. One respondent described this, drawing on their experience providing feedback as part of a research study:

“[W]e had a number of people write in and say ‘Participating in a study is too much for me, I see my scores going down week by week, I see that my medicine is not making any difference, this is a lot’, ‘having access to my data’ – because we gave our participants access to their data – ‘having access to my data is just depressing me; having the ability to do some things now and knowing that I’m not going to have the ability to do them later, is crushing me, and being reminded of this three times a day is overwhelming, I have to quit’, and so that was some very sobering feedback (Staff member, non-profit organisation)

A researcher from a company similarly described how users of their tool will ‘often … reach out and say, like, hey, I want to talk to somebody about this’. The concerns that these respondents highlight, in which people feel overwhelmed and emotionally affected by an intimate, one-to-one encounter with data, both encapsulates the threatening nature of the data shadow and draws discussion back towards that of the bioethical discourses with which we started. As another researcher at a non-profit described, this relationship revolves around a core dilemma

“In terms of detection, if you tell them that they are at increased risk. OK, you’ve told me this. What can I do then to reduce that risk? And obviously in this space we can’t really do that. We can just say there are some risk factors that have been associated, but we can tell you how to reduce your risk by 20 percent or something like that. Can you cope with that? … So, it’s quite a hard topic to discuss with them.” (Staff member, non-profit organization)

As this extract highlights, and as introduced earlier, discussion around the ‘data encounter’ in Alzheimer’s disease – and with risk information collected from genetics, family history, or lifestyle factors – has long revolved around the potential for psychological harm in the absence of an effective therapy (Brayne and Kelly, 2019; Lock, 2013; Post, 1996). This threat persists even as developers attempt to establish an ‘enabling’ data relationship – and, as this respondent describes, remains a ‘hard topic’, despite increasingly well-established clinical approaches to risk communication (e.g. Harkins et al., 2015). Indeed, some respondents suggested that this potential for harm may be intensified in the context of cognitive and behavioural data that are seen as ‘a bit of your identity’ in a way that biological markers are not (Digital health researcher). Risk and its communication serves as an engine for scientific and commercial development (cf Dumit, 2012; Milne, 2018), for example, in the case of pharmaceutical innovation or insurance markets. However, for some, the threat posed by the encounter with the Alzheimer’s data shadow in this context has emerged as a barrier to their research and development:

“the first day that we pitched the idea in [X] we had one of the major investors in [X] went on a crusade to stop us from developing our technology, and we’ve had that over the last seven years as well, keep coming back at us is if there’s no treatment there’s no point in diagnosis, and what you do is unethical. I think I’ve expressed that enough and I still believe as a researcher, as a patient, that’s not the right approach but it is a stance some people take. (CEO, UK digital health company)

The intensity of concerns around risk, and the ability of these concerns to shape the development of digital tools, is a prominent feature of Alzheimer’s disease. It characterises scientific, clinical and popular discussions of early detection and risk prediction in a way that can become forceful in technological development, as previously in the case of pharmacogenetics and biomarker development (Boenink, 2018; Hedgecoe, 2008). In the case of digital phenotyping, researchers described the problem of risk communication as ‘one of the kind of big ethical concerns’ (company researcher), which, for their company, currently precludes the possibility of any direct relationship between the individual and their data shadow. Further, the menace posed by this relationship re-emphasises the need for digital phenotyping to capture and be embedded in everyday life, to ‘appropriately support people through this’ (company researcher). Such considerations emphasise that, for individuals and organisations, data shadows cast long into the future, and living with them is a long-term commitment.

## Working with shadows

In the preceding sections, we have introduced two dominant threads in how the relationship between data subjects and their data selves is conceptualised by researchers and developers working in the field of digital tools for the early detection of Alzheimer’s disease. We have used the metaphor of the data shadow to explore two elements of digital phenotyping and its products in this context – their representational function and their role as enabling or threatening materials.

For our interviewees – personally, scientifically or financially invested in the futures of digital health – digital phenotyping approaches draw from the wider promise of data the possibility of a different way of understanding and representing the ageing brain. For these researchers, as for advocates of digital psychiatry such as Insel, the potential of the data shadows of Alzheimer’s disease is that of an individualised, longitudinal and ‘ecological’ mode of representation. The construction of this way of seeing illustrates how the representational apparatus of Alzheimer’s disease is being assembled and which aspects of the disease are being exposed to, or concealed from, view. It also, however, shows how this process reflects and reproduces wider discourses around the possibilities and perils associated with inferences made through our digital traces. Thus, our respondents highlight how digital phenotyping and the elaboration of digital data shadows intersects with existing practices of data collection and analysis, as well as the ontological and epistemological commitments of contemporary Alzheimer’s disease research. Participants draw attention to the potential for complementarity but also tensions between digital data and those that emphasise biology, biomarkers and the therapeutic models that target them. These relations further gesture towards the commercial arrangements and health system architectures associated with different ways of seeing, and emphasise the persistent forms of strategic relationality through which data shadows are cast, bodies made visible and visualisable or left in obscurity (cf Leonelli et al., 2017).

In addition, however, in our respondents’ discussions of digital phenotyping the ‘data shadow’ is more than representational, playing a material role in the emergence and promise of new technologies and tools. The Alzheimer’s disease data shadow has the potential to be materially forceful both in the lives of data subjects and to empower and enable – but also to threaten. For many of our respondents, this is captured in both the opportunities the data shadow affords for ‘self-care’ and the facilitative role that the data shadow can play as it stands in for the data subject in their interactions with healthcare and insurance. Conversely, our respondents highlight the threat this shadow poses to the individuals living ‘in the shadow’ of future dementia, connecting emerging discussions around digital phenotyping with longstanding bioethical debates (Karlawish, 2011; Post, 1996). This threat looms large in a field in which the question of ‘would you want to know’ is a recurrent feature of popular and policy discussions around the early detection of disease. For our respondents, this threat is to both those living with such shadows, and to those looking to establish a space for diagnostic innovation and negotiate the challenges of ‘innovating with care’ in Alzheimer’s disease (Boenink et al., 2016). Further, the potential to enable – within a particular political and economic configuration of preventative medicine – also suggests the potential challenges associated with the absence of data, or living without a data shadow as digital phenotyping practices become embedded in healthcare or insurance markets.

The ability of data shadows to empower, enable and threaten, and the consequences of their absence, draws particular attention to the way shadows are situated in time. As our respondents described, digital phenotyping tools for early detection do not necessarily indicate the current state of the brain, instead creating data shadows that are cast forward in time. In the context of research that is moving away from a focus on symptomatic diagnosis of disease towards early detection and the identification of the risk of future dementia, analyses of digital traces are a means of knowing both the current and (possible) *future* self.

These considerations point to the emerging complexities of digital phenotyping as developers attempt to integrate the collection of digital ‘biomarkers’ by active or passive means into existing clinical domains. Our use of the data shadow to explore these themes, and their representational and material articulations, points to the ways in which digital phenotyping requires continued attention to inter-twined ethical and epistemological considerations. The empowering, enabling and threatening aspects of the shadow highlight the challenges associated with controlling or containing the Alzheimer’s disease data self. In addition, they suggest the importance of continuing to revisit what is, and what is not, made visible in the process of representing the ageing brain, what is gained or lost with the commitment to longitudinal, situated representation, and what features of ageing continue to remain unseen.
